# Child, neglect and oral health

**DOI:** 10.1186/1471-2431-13-188

**Published:** 2013-11-18

**Authors:** Caroline Barbosa Lourenço, Maria Vieira de Lima Saintrain, Anya Pimentel Gomes Fernandes Vieira

**Affiliations:** 1 Private Practice, Fortaleza, Brazil; 2 Public Health Master Program, University of Fortaleza (UNIFOR), Fortaleza, Brazil; 3 Family Health Master Program, Fundação Oswaldo Cruz (FIOCRUZ), Fortaleza, Brazil

**Keywords:** Neglect, Oral health, Maternal behavior

## Abstract

**Background:**

Despite advancements in oral health policies, dental caries still a problem. The lack of parents/caregiver’s care regarding child’s oral health, which characterizes neglect, may lead to a high prevalence of caries. Therefore, the objective of this study was to analyze the relation between dental caries and neglect in five year-old children.

**Methods:**

Quantitative study performed in two different moments. First, the children underwent oral examinations and physical inspection. Then, a semi-structured interview was performed with parents of children with high and low caries rate.

**Results:**

In all, 149 physical inspections and oral exams were performed. The number of decayed, missing and filled teeth – dmf-t was 2.75 (SD 2.83); 16 children had extremely high values (dmf-t ≥7), 85 intermediate values (1 ≤ dmf-t ≥ 6) and 48 extremely low (dmf-t = 0). Nearly all caregivers were female (96.7%; n = 29), mostly mothers (93.3%; n = 28). Associations were found between caries experience and reason of the last consultation (p = 0.011), decayed teeth and child’s oral health perception (p = 0.001). There was a trend towards a significant association between general health and decayed teeth (p = 0.079), general hygiene and caries experience (p = 0.083), and caries experience and number of times the child brushes the teeth (p = 0.086).

**Conclusion:**

There’s a relation between caries experience and children’s oral health perception by caregivers, as well as between caries experience and children’s access to dental care. There is a trend towards association between caries experience and risk factors suggestive of neglect.

## Background

The United Nations Convention on the Rights of the Child declares, in the article 27th, that “States Parties recognize the right of every child to a standard of living adequate for the child’s physical, mental, spiritual, moral and social development”. It also states that “the parent(s), or others responsible for the child, have the primary responsibility to secure, within their abilities and financial capacities, the conditions of living necessary for the child’s development” [1].

However, despite the great advancements in oral health policies, caries disease is still a severe problem that hinders such development [2]. Among the factors associated with the high prevalence of caries in infants is the lack of caregiver’s care concerning child’s oral health [2,3], which characterizes a neglect act [4]. Neglect is “a type of maltreatment that refers to the failure by the caregiver to provide needed, age-appropriate care although financially able to do so or offered financial or other means to do so” [5]. Neglect can be subdivided, being dental neglect, a form of physical neglect. According to the American Academy of Pediatric Dentistry, dental neglect is willful failure of parent or guardian to seek and follow through with treatment necessary to ensure a level of oral heath essential for adequate function and freedom from pain and infection [6].

Yet, despite the potential association between the prevalence of dental caries and neglect, little is known about the relation between them, which is the main objective of this research.

Caries disease is probably the most prevalent of all child diseases, and, if left untreated, it may cause many problems such as pain, suffering, productivity loss – for instance, at school – and development of severe functional and social limitations in the individuals afflicted by it [7,8]. Children are considerably dependent on their parents, not least in relation to achieving good oral health [9]. It is important to highlight that children who are up to the age of seven do not have manual ability to brush their teeth without an adult’s supervision, as well as they do not have autonomy to access dental services. “The person responsible for the children and adolescents’ health has to be engaged in the treatment in order to obtain a positive result” [7]. Thus, caregivers must get information and provide the child with preventive and restorative care.

The 49th World Health Assembly, in 1994, declared violence as one of the main world public health problems [10]. Analyzing the nature of violent acts, the WHO [10] classified it into four types: physical, sexual, psychological and involving deprivation or neglect. Among the social groups that are more vulnerable to violence, children represent the most vulnerable one. Regarding the types of violence against children, studies show the prevalence of neglect in relation to others [4,11,12].

In the 1990’s, the WHO and the United Nations Children’s Fund (UNICEF) developed the Integrated Management of Childhood Illness (IMCI) as the main strategy to improve the quality in children’s health care. This strategy includes contents for evaluation, classification and treatment of diseases and health problems [13]. In 2003, the Pan American Health Organization (PAHO) incorporated the component “Child Abuse” in the IMCI strategy. According to IMCI, neglect refers to “the failure of a parent to provide for the development of the child, with or without intention, in one or more of the following areas: health, education, emotional development, nutrition, shelter and safe living conditions, being the abandonment considered the worst neglect level”. The investigation on children’s care in relation to hygiene is an important guide to determine the level of interest of parents and caregivers, and to do so, it is indispensable to observe cleanliness/general aspects of the hair, nails and teeth. Besides that, a delayed consultation in the healthcare service may represent an attitude that shows little interest in child’s needs or affections. It is important to say that the delay may occur due to parents’ intention, who might want to omit a lesion they may have caused or prolong child’s pain [13]. According to the World Report on violence and health, there are many manifestations of child neglect, including non-compliance with health care recommendations, failure to seek appropriate health care, deprivation of food resulting in hunger, and the failure of a child to thrive. In addition, abandonment, inadequate supervision, poor hygiene and being deprived of an education have all been considered as evidence of neglect [10].

In dentistry, child neglect act is manifested in several ways: lack of caregivers’ interest in acquisition of information related to dental care, lack of preventive care that shall be performed at home by caregivers (e.g. oral hygiene), dental appointment no-shows, among others. Thus, it is necessary to evaluate the association between neglect and oral health problems in children.

While many people believe that children’s oral health problems boil down to the lack of access to oral healthcare services in the public health system, it can be observed that in some cities, with a good structured free public system attending 100% of the population (e.g., Pacoti-Brazil), the demand and utilization of such service is below children’s needs [14-17]. Pacoti won the Smiling Brazil Prize for best performance in oral health care in Ceará State in the year 2005; and the Unicef Seal (related to excellence in education, health and social security) in 2000, 2002, 2004, 2006 and 2008, thus attesting the quality of the public (free of charge) dental service provided to its population. Its schools perform activities such as supervised tooth brushing, fluorine use and educative lectures, and children receive scheduled assistance for restorative treatment. Still, despite all those efforts, there is a low demand and utilization of children’s individual prevention and rehabilitation oral health services in this city. The reason for that is hard to understand, and one can question whether it occurs due to caregivers’ neglect.

Thus, this study aimed to analyze the relation between dental caries indexes and the occurrence of five-year-old child neglect by parents. That age group was set due to the interest in the deciduous teeth, and the fact that this age is used as reference for caries epidemiological research [18]. Additionally, children at that age were already born in a city with a well-structured public dental service and should not have had any problems related to access to oral health prevention and promotion actions, as well as to restorative care. At that age, children do not have enough motor ability to perform oral hygiene (e.g., tooth brushing, flossing), and do not have autonomy to access dental services, which means they are totally dependent of their caregivers. Therefore, it is possible to evaluate the influence of child neglect by caregivers in a more precise manner within this age group.

## Methods

This is a quantitative study conducted in two stages. First, all 5 year-old children from Pacoti city underwent oral examinations according to the criteria of SBBRASIL Project (a national oral health epidemiological survey), and physical inspection, according to criteria of the Detection and Prevention of Child Abuse, a component of the Integrated Management of Childhood Illness (IMCI) [13]. Before the examinations, all children underwent a supervised tooth brushing activity. The intraoral exam was performed using a flat surface dental mirror and tongue depressors under natural light. Initial stages of dental caries such as white spots were not taken into account, according to the criteria used in the SBBRASIL project [19]. It was considered decayed, the teeth with coronal cavities, sulcus cavities, fissures or smooth surface presenting cavity or enamel discoloration or having temporary restoration (except glass ionomers) [19]. In addition, these children underwent physical inspection in order to observe any signs of neglect. The inspection verified: general cleanliness, hair, nails, bruises/wounds on skin and occurrence of physical or mental disability. The place where the examinations took place was breezy and properly lit and there was a water supply system nearby. Only one trained examiner, who was submitted to the intra-examiner calibration with kappa > 0.8, evaluated the children. The children who refused to participate in the research (oral examination and physical inspection), even with parental consent, were not included in the research.

In the second stage, an interview was performed with the caregivers of children who presented extremely high and low number of decayed, missing (due to carious lesion or with indicated extraction) and filled teeth (dmf-t) based on a semi-structured questionnaire regarding their perception concerning the children’s oral health, risk factors for caries, suggestive signs of neglect and children’s access to dental care. The data were organized and grouped according to the study aims and analyzed using the statistics program SPSS 15.0 for Windows (SPSS Inc., Chicago II, USA). They were described and correlated with the children’s dmf-t index.

The legal guardian of all children participating in the research signed a Consent Form. The project was approved by the Ethics Committee of UNIFOR - No. 156/2011.

## Results

Between September and December of 2010, 149 children, of a total of 174 children in the municipality, underwent oral and physical exams. Twenty-three parents/caregivers refused to sign a consent form to participate in the research, as well as two children did not accept to undergo the oral and physical examination. Among the analyzed children, 67 studied in urban day care schools and 82 in rural area schools. It’s worth saying that all 5-year-old children from the city were registered for school, however, some of them did not attend classes regularly. These children were examined at their homes.

In the oral exam, the average dmf-t was 2.75 (±2.83). In all, 16 children showed extremely high values (dmf-t ≥ 7), 85 presented intermediate-high values (1 ≤ dmf-t ≥ 6) and 48 low values (dmf-t = 0). Thus, 32.2% of the examined children were caries-free individuals. Considering the components related to the caries experience index (dmf-t) of the 149 examined children, there were 328 decayed teeth (78.85%), 65 teeth with extraction indication (15.62%) and 23 filled teeth (5.5%), besides the 2545 healthy teeth and 53 missing ones. There was an average per individual of 2.20 (±2.41) decayed teeth, 0.44 (±0.93) teeth with extraction indication and 0.15 (±0.59) filled teeth, which shows a greater importance of the components decayed and extraction indication in relation to the filled teeth component.

In the physical exam, concerning the aspects related to hygiene care (e.g., cleanliness, teeth, hair, nails), only one child presented poor hair and nails general hygiene and 48 (32.2%) poor teeth hygiene. Regarding the occurrence of physical lesion, only one child presented wounds in the arms and concerning developmental delays, only one child, with cerebral palsy, presented it (Table 1).

**Table 1:** Aspects related to children’s care (n = 148) in hygiene, lesion occurrence and developmental delay – Pacoti, 2010

**HYGIENE CARE**	**n**	**%**
**Poor General Hygiene**	1	1.5
Hair	1	1.5
Nails	1	1.5
Teeth	48	32.2
**Physical Lesion**	**n**	**%**
Wounds (in the arms)	1	1.5
**DEVELOPMENTAL DELAY**	**n**	**%**
Brain Palsy	1	1.5

In the second stage of the research, 16 caregivers of children with the best caries index values (dmf-t = 0) and 14 caregivers of the children with the worst values (dmf-t ≥ 7) were interviewed. Two caregivers of children with the worst values did not participate in the study because they moved to other cities.

Nearly all caregivers were female (96.7%; n = 29), mostly mothers (93.3%; n = 28). Regarding marital status, most had a domestic partnership (46.7%; n = 14), seven (23.3%) were married, six (20%) single, two (6.7%) divorced, and one (3.3%) widowed. The interviewees presented the following education level: 60% (n = 18) had studied for less than nine years; 3.3% (n = 1) for nine years; 3.3% (n = 1) between 9 and 12 years – incomplete high school; 23.3% (n = 7) for 12 years –high school degree; 3.3% (n = 1) had an incomplete undergraduate course and 6.7% (n = 2) had a completed undergraduate course. With regards to family income and profession, most (83.3%; n = 25) received up to US$ 315; 11 (36.7%) were agriculturists; 13 (43.3%) housewives; three (10%) public servants; one (3.3%) retired, and two (6.7%) had other jobs. It’s worth saying that in most of the visited houses (70%; n = 21) the inhabitants were a family unit (a father, a mother and their children). Concerning the frequency of alcohol and tobacco use, 86.7% reported never using alcohol and 93.3% did not smoke. The frequency of alcohol and tobacco use by other people living in the house was asked, most interviewed individuals (83.3%; n = 25) answered that nobody smoked, 20 (71.4%) never used alcohol and only two (7.1%) used alcohol four or more times a week (Table 2).

**Table 2:** Demographic socioeconomic status of the interviewed individuals and risk factors – Pacoti, 2010

**INTERVIEWEE’S GENDER**	**n**	**%**
Male	1	3.3
Female	29	96.7
**MAIN CHILD’S CAREGIVER**	**n**	**%**
Mother	28	93.3
Grandparents	2	6.7
**INTERVIEWEE’S EDUCATIONAL PROFILE**	**n**	**%**
Incomplete Elementary School	18	60
Complete Elementary School	1	3.3
Incomplete High School	1	3.3
Complete High School	7	23.3
Incomplete Higher Education	1	3.3
Complete Higher Education	2	6.7
**FAMILY INCOME**	**n**	**%**
Up to 1 minimum wage (MW) – R$545,00	25	83.3
Between 1 and 2 MW – R$546,00 a R$ 1090,00	2	6.7
Between 2 and 3 MW – R$1091,00 a R$1635,00	2	6.7
3 or more MW – R$1636,00	1	3.3
**RESIDENTS**	**n**	**%**
Father, mother and children	21	70
Mother and children	4	13.3
Parents and relatives	4	13.3
Other	1	3.3
**INTERVIEWEE’S HEALTH PROBLEMS**	**n**	**%**
Yes	5	16.7
No	25	83.3
**FREQUENCY OF ALCOHOL USE OF THE INTERVIEWEE**	**n**	**%**
Never	26	86.7
Up to 2X	4	13.3
**FREQUENCY – TOBACCO USE BY THE INTERVIEWEE**	**n**	**%**
No	28	93.3
Yes	1	3.3
Occasionally	1	3.3

Concerning whether the child had already been to the dentist or not, 16 (53.3%) interviewees answered *yes* and 14 (46.7%) answered *no*. Among the interviewees who answered *no*, 71.4% (n = 10) said that they did not take the child to the dentist because they thought there was no need to do so; and, among the ones who answered *yes*, six took the child to the dentist for a check-up, three because of pain/abscess, two due to caries, two because of dental fracture (dental trauma) and three for other reasons. Regarding the number of times the child brushes the teeth a day, 10% (n = 3) reported brushing once a day; 30% (n = 9), twice a day; 56.7% (n = 17), three times a day; and 3.3% (n = 1), did not know. When they were asked about whether oral health affects the child’s relationship at school and at home, two (6.6%) and four (13.3%) people answered *yes*, respectively. Regarding the occurrence of discrepancy between the aspect of the child and of the interviewee, there was one positive case (Table 3).

**Table 3:** Oral hygiene guidance during prenatal, child’s oral health perception by caregivers and discrepancy between the hygiene aspect of the child and caregiver – Pacoti, 2010

**ORAL HYGIENE GUIDANCE DURING PRENATAL**	**n**	**%**
Yes	7	23.3
No	23	76.7
**ORAL HEALTH AFFECTS CHILD’S RELATIONSHIP AT SCHOOL**	**n**	**%**
Yes	2	6.67
No	28	93.3
**ORAL HEALTH AFFECTS CHILD’S RELATIONSHIP AT HOME**	**n**	**%**
Yes	4	13.3
No	26	86.7
**DISCREPANCY BETWEEN THE ASPECT OF THE CHILD AND INTERVIEWEE**	**n**	**%**
Yes	1	3.3
No	29	96.7

In order to analyze the relation among the dmf-t index; caregiver’s perception over their children’s oral health; access to dental care services by five-year-old children and risk factors and suggestive changes of neglect, the corresponding variables and such aspects were crossed. Significant associations were found between the caries experience index (dmf-t) and the reason for the last consultation (p = 0.011). Regarding the continuous variables, there were significant associations between the number of decayed teeth and the number of children in the family (p = 0.005), number of decayed teeth and child’s oral health status given by the interviewee (p = 0.001), number of healthy teeth and number of children in the family (p = 0.001), among others (Table 4).

**Table 4:** Associations between continuous variables relationship a number of decayed teeth, caries index, child’s oral health status according to the caregiver and number of children

**ASSOCIATIONS**	**r**_**s**_	**p**
Number of decayed teeth X Number of children	0.503	0.005
Number of decayed teeth X Child’s oral health status according to the caregiver	−0.670	>0.001
Number of teeth with extraction indication X Number of children	0.404	0.027
Number of teeth with extraction indication X Child’s oral health status according to the caregiver	−0.478	0.009
Caries index X Number of children	0.536	0.002
Caries index X Child’s oral health status according to the caregiver	−0.657	>0.001

In order to verify if children’s of working caregivers had more dental caries than those of non-working caregivers, Mann Whitney test was performed. The findings reveal that there is no dmf-t difference in children with and without working caregivers/mothers (p > 0.05). However, there was a difference between the number of teeth indicated to extraction among the two groups (working and non-working caregivers), where the children from the working caregivers presented less teeth needing to be extracted than those of non-working caregivers (p = 0.047). Qui-square test also reviewed no relationship between working caregiver and dental care access (visit to the dentist) – p > 0.05.

It was verified a *trend towards a significant association* (0.05 < p > 0.1), when performing the Kruskal-Wallis’ test, between general hygiene and number of decayed teeth (p = 0.079), general health and caries experience index (p = 0.083), caries experience index and number of time the child brushes the teeth (p = 0.086), caries experience index and whether oral health affects the child’s relationship at school (p = 0.077). A *trend towards a significant association* was also verified, through the Mann-Whitney’s test, between caries index and occurrence of discrepancy between the aspect of the caregiver and the aspect of the child (p = 0.087).

## Discussion

According to authors’ best knowledge, this was the first time that children’s oral health neglect was studied in a city where a good and public (free of charge) oral health care system is available to everyone. Thus, it was possible to evaluate more accurately the influence of children’s oral health neglect by caregivers.

According to the California Society of Pediatric Dentists, a parent can only be considered negligent and proper intervention carried out, after it has been adequately alerted by a health care professional regarding the nature and extent of the child’s condition, the specific treatment needed, and the mechanism of accessing that treatment [20]. Despite this understanding, it was possible to identify important factors related to neglect that can be discussed in this study, such as: oral health influence on child’s relationship at school and within their family; associations between general hygiene and caries experience, number of children and caries experience, caries experience and child’s oral health status according to caregiver, caries experience and the number of times a child brushes its teeth, caries experience and the reason of the last dental consultation.

Regarding the number of times a child brushes its teeth a day, it was found a *trend towards a significant association* with caries experience. Children who brush the teeth more than once a day with fluoride dentifrice have a better caries reduction than those who brush fewer times [21]; what corroborates with the findings of the present study. It’s worth saying that the World Health Organization, in its world report on violence and health, highlights the non-compliance with the health care recommendations and inadequate supervision as evidence of child’s neglect [10].

A high number of caregivers of the current study reported not taking the child to the dentist because they thought it was not necessary. Unfortunately, it is not uncommon to see parents giving low priority to health-promoting efforts in the form of regular dental check-ups for their children, which puts them in a risk group for dental neglect. However, this reality is not uniform around the globe. A study carried out in Greece found out that 95% of parents thought that a child should visit the dentist at an early age and 79.5% of responders answered that they had already visited the dentist with their child for different reasons [22].

The failure to seek appropriate health care, according to the World Health Organization [10], is also a manifestation of neglect. Early dental care reduces the chances of a child to develop oral diseases, especially dental caries. It also familiarizes the child with the environment and the professional, creating opportunities to acquire healthier habits and, consequently, a good quality of life [23].

It was observed that many caregivers only seek dental care when the child feels pain/abscess, contributing, then, for a high caries experience. The prevalence of toothache tends to be inferior among children who had been to the dentist at least once in their life and those who had been to the dentist in the last year [24]. When a child goes to the dentist only when there’s pain/abscess, the treatment is often more radical and sometimes it is necessary a tooth extraction. Early deciduous tooth loss may cause many occlusion problems, the most common being space loss, antagonist tooth extrusion, adjacent teeth rotated or misplaced, besides atypical deglutition and phonation problems. It stands out the fact that all these disorders will need a high-cost specialized treatment, which could be avoided with simple and low-cost preventive measures.

Regarding caregivers’ perception concerning to their children’s oral health, there were significant associations between number of decayed teeth and child’s oral health status according to the interviewee; this shows that, although parents are aware of their children oral health conditions, many of them have never taken their child to the dentist. It is interesting to point out that, children from working caregivers did not had higher prevalence of dental decay. The poor structure of oral services can be pointed out as one of the factors responsible for caries and tooth loss in children [25]. Adding up to this, there’s the fact that mothers in Northeast part of Brazil (one of the poorest areas in the country) want to provide their children with dental treatment but they can’t afford it. On the other hand, there are studies that verified the depreciation, by caregivers, of the deciduous dentition, predominant in children, due to the fact that it is a temporary tooth [26]. Corroborating with this last statement, Siqueira *et al.,*[2] reported, in a research involving 4 and 5-year-old children, the high rate of dental appointments absence (42.4%) and concluded that this fact indicates either the unawareness of the importance of the treatment or health neglect. They also found that most of the absences were by poor families that, even with free treatment, were not interested in attending dental appointments.

In Pacoti, according to the oral health coordination, there’s a usual complaint by dentists regarding patients’ absence to appointments, both by adults and children, even in urban areas, where commuting is easier. It is known that the way people notice the health-disease process influences, directly, on their actions and oral health care [27]. “Individuals’ beliefs and behavioral patterns are part of the health care system and they come, mostly, from cultural rules” [28]. Parents and caregivers still think that caries cannot be avoided and they see it as a natural phenomenon, not as a disease [27]. In this study, none of the interviewed individuals reported having difficulties to access dental services as a way to justify their attitude for never taking the children to the dentist. In the case of Pacoti, the dental services are universal and free of charge for all patients. The public health system develops actions on public health promotion, prevention and rehabilitation (e.g., including individual’s dental services). The public health units are located in several points of the city and of easy access to the whole population. Therefore, despite the theoretical dependency on services’ availability and affordability, the parents/caregivers in Pacoti do not have such limitations. That is one of the main reasons that the city of Pacoti was chosen for this study.

The majority of caregivers justified dental treatment absence to lack of interest, even though they knew that the child’s oral health status was not adequate. Therefore, the apparent neglect of caregivers regarding children’s oral health care may be partially explained by a low educational and social level, the believe that dental decay is a natural phenomenon and that deciduous teeth are not important for the child general health.

Families living in disadvantaged circumstances may experience greater levels of stress, isolation and family conflict. Parents may have more feelings of powerlessness to achieve good oral health for their children. These factors may have an indirect effect on disease through their influence on diet and behavior, for example, and must be taken into account if strategies for managing oral health care are to succeed [29].

Three-day dietary diaries are commonly used by pediatric dentists to assess risk factors linked to diet. However, in community based studies, this diaries are difficult to be done in an accurate manner, specially because diet is a modifiable risk factor. According to Chankanka, some dietary factors are associated with caries at one age only, while others are associated with caries across childhood. He concludes there is a complex relationship between diet and caries, and that more studies are needed in the field, including studies that place more emphasis on investigation of modifiable risk factors [30]. Therefore, in the present study, no attempts were made to gather information regarding children diet.

A curious datum found was the significant association between general hygiene and caries experience, showing that when there is child’s general health neglect, such as absence of hair and nails cleanliness, there is also oral hygiene neglect.

It is important to mention that the reporting, by health professionals, of suspected child abuse and neglect is mandatory by law in various countries, including Brazil [10]. Neglect evidences, regarding oral and general health, must be treated as soon as they appear [10]. In order to do so, primary care professionals have a decisive role facing this problem, once they have a close social interaction with the community. Preschool and school age children can be provided general and oral health guidance through educative and preventive measures [23]. Nevertheless, for a better result, it is important to expand such actions to the whole family.

Within this dimension, health promotion actions must be performed through an accessible scientific language, considering the references and experiences in the community. Information is the basis for behavior change, but, by itself, it is not enough. Thus, it’s expected that aspects such as responsibility and self-care, as well as the understanding of rights and duties by the individual as a co-responsible subject in its health-disease process, can be gradually learned/internalized and ignite changes in caregivers and also in children, as demonstrated by Nammontri *et al*. [31]. The city of Pacoti develops health promotion and prevention activities with school age children and pregnant women. At school, supervised brushing and fluoride use are performed weekly; educative speeches are performed every three months. All of those activities are also free of charge for the population.

A trend towards a significant association was verified between the effect of oral health on child’s relationships at school and at home. Many caregivers reported that their children receive pejorative nicknames because of caries tooth destruction. It stands out that violence also consists in actions that affect moral, mental and spiritual integrity [32]. The child’s relationship at school and within their family produce impacts on self-esteem construction. This, consequently, interferes in identity construction and generates reflexes in the individual’s relationship with the world. Low self-esteem becomes a risk factor for physical and mental health, and it can make children develop feelings such as anguish, suffering, gloom, shame and other bad thoughts. Thus, it is important to keep a positive self-esteem for children’s growth and development [33]. That is, although parents are aware of the humiliating situation their children have been through, which can cause among other problems, a low self-esteem, they do not try to solve such problem, what can be characterized as violence act against the child.

The main purpose of this article was to scientifically investigate the belief that neglect (including dental neglect) influences children oral health. According to the author best knowledge, this issue was properly investigated in this research and therefore needs to be shared to the scientific community, specially because it is the first time that such relationship is evaluated in a municipally where access to dental treatment doesn’t seems to be an issue (all residents have quality health units close to home and free of charge).

Lastly, but not less importantly, two points need to be raised regarding this study. First, the utilization of the expression ‘trend towards a significant association’, which was used when the p value was between 0.05 and 0.1. Several authors have used this interpretation [34-36], understanding that some flexibility is desirable in interpreting p values, and that calling any value with p > 0.05 “not significant” is not recommended, as it may obscure results that are not quite statistically significant but do suggest a real effect [37,38]. Nevertheless, the authors of the present study, which agree with the understanding just described, also appreciate that these trends towards significance need to be seen with responsiveness and precaution. Secondly, despite knowing that in Pacoti the dental services are universal and free of charge for all patients, and that could mean complete access to dental service by the whole population, the authors appreciate that other factors, such as cultural and socioeconomic issues influence access. Therefore, some of these issues, few already discussed in this section, deserve further investigation.

## Conclusion

Neglect evidences, regarding oral and general health in children, must be treated as soon as they appear. The reporting, by health professionals, of suspected child abuse and neglect is mandated by law in various countries, including Brazil. However, in order to do so, the health professional needs to understand what is dental neglect, as well as its risks factors and variables associated with it.

In conclusion, this study showed a relation between caries experience and children’s oral health perception by caregivers, as well as between caries experience and children’s access to dental care. There is a trend towards association between caries experience and risk factors suggestive of neglect. Knowing the complexity of the theme studied in this research, it is not the authors’ intent to answer all questions regarding this issue. On the other hand, it is believed that the findings of this research contribute to future studies involving other places with good, free of charge, dental care services structure, so that they can help clarify and indicate solutions for the relationship between child’s oral health and neglect.

The detection of child’s neglect by parents is, most of the time, a difficult task. There are few studies about it. However, some factors that indicate neglect can and must be identified as was done in the present study. This is a way to prevent neglect from being ignored, jeopardizing the child’s quality of life. This is one of the main contributions of this study to the contemporaneous literature.

## Competing interests

None of the authors have received, in the last five years, reimbursements, fees, funding, or salary from an organization that may in any way gain or lose financially from the publication of this manuscript. Oswaldo Cruz Foudation, where the co-author APGFV works, is responsible for this article-processing charge. None of the authors hold or have the intention to hold stocks or shares in an organization that may, in any way, gain or lose financially from the publication of this manuscript. None of the authors hold or is applying for a patent related to the content of this manuscript, nor have received reimbursements, fees, funding, or salary from an organization that holds or has applied for patents related to the content of this manuscript. The authors do not have non-financial competing interests (political, personal, religious, ideological, academic, intellectual, commercial or any other) to declare in relation to this manuscript.

## Authors’ contributions

APGFV and MVLS were responsible for the conception of the study design. CBL was responsible for data collection and organization. All authors, who were also responsible for the draft of the manuscript, performed data analysis and interpretation. All authors read and approved the final manuscript.

## Pre-publication history

The pre-publication history for this paper can be accessed here:

http://www.biomedcentral.com/1471-2431/13/188/prepub
